# Virtual Reality for Upper Extremity Rehabilitation—A Prospective Pilot Study

**DOI:** 10.3390/healthcare11101498

**Published:** 2023-05-21

**Authors:** Pinar Tokgöz, Dirk Wähnert, Andreas Elsner, Thomas Schack, Miguel Angel Cienfuegos Tellez, Jens Conrad, Thomas Vordemvenne, Christoph Dockweiler

**Affiliations:** 1 Digital Public Health, Department Digital Biomedicine and Health Sciences, School of Life Sciences, University of Siegen, 57076 Siegen, Germany; 2 Clinic for Trauma Surgery and Orthopedics, Protestant Hospital of Bethel Foundation, University Hospital OWL, 33617 Bielefeld, Germany; 3 German Institute for Orthopedics, Osteopathy and Sports Medicine (DIOSS), 33604 Bielefeld, Germany; 4 Neurocognition and Action Research Group, Faculty of Psychology and Sports Science, University Bielefeld, 33615 Bielefeld, Germany; 5 Center for Outpatient Rehabilitation, 33617 Bielefeld, Germany

**Keywords:** rehabilitation, virtual reality, upper extremity, virtual rehabilitation, pilot study

## Abstract

Applications related to virtual reality are a rapidly growing area. Thus, these technologies are also increasingly used in the field of medicine and rehabilitation. The primary objective of this prospective pilot study was to investigate the feasibility, user experience and acceptance of a virtual-reality-based system for upper extremity rehabilitation. The study was conducted as a single-center trial over 16 weeks. The eligibility criteria included rehabilitants with upper extremity injuries of at least 18 years of age who were fluent in spoken and written German. After detailed instruction, each participant was asked to complete daily 30 min exercises over 15 training sessions with the virtual reality system consisting of three different training modules. Outcomes were assessed pre-study and post-study using standardized clinical measures. In addition, qualitative interviews with rehabilitants as well as therapists regarding user experience and acceptance were conducted. Six participants were recruited for the pilot study, of which five underwent virtual-reality-based rehabilitation. Overall, the clinical measures showed a positive tendency over the course of the study, even if the results were not significant. Furthermore, the virtual-reality-based training was well accepted by the participants as well as therapists. Given these findings, it will be beneficial to evaluate virtual reality for rehabilitation in further research.

## 1. Introduction

In 2018, diseases of musculoskeletal systems were the most frequent reasons for outpatient rehabilitation among women (74%) and men (67%) in Germany [1]. Most of the complaints affected the upper extremities [2]. Hence, rehabilitation is of high importance for recovery of people suffering from upper extremity injuries and the maintenance of joint function [3]. 

The dissemination of technological innovations has opened up new possibilities for delivering rehabilitation services. A major technological innovation is virtual reality (VR) [4]. VR in the field of medicine emerged as a new therapeutic tool not only for medicine but also for the treatment and rehabilitation of people with disabilities and injuries. Rehabilitation through VR describes an assistive health technology that is used to recover motor or sensory skills lost due to accident or illness through a virtual but interactive environment [5]. Combined with three-dimensional motion analyses, these systems have great potential for musculoskeletal rehabilitation, as they present a cost-effective complementary treatment to traditional rehabilitation and, in line with traditional rehabilitation, enable personalized treatment, increasing their compliance as well as self-management and functional recovery [6]. By performing intensive repetitive-task-based treatment, VR can be utilized to collect real-time data to track progress during rehabilitation both for rehabilitants and therapists [7]. In this context, the therapist might be able to measure and control various responses provided by users [8]. Moreover, VR allows continuous monitoring of several rehabilitants at the same time, saving time and cost [9]. Additionally, VR makes it possible for rehabilitants to perform therapy in the comfort of their own home, especially when they are not able to attend outpatient rehabilitation centers regularly [10,11]. VR-based systems might be advantageous not only for those who live far from rehabilitation centers, but also for people with severe disabilities restricting their mobility [12]. The main characteristics of VR are controlling interactions and enriching the person’s experience. Indeed, VR is very flexible and programmable. The applications of VR in the medical field are numerous and a high number of research studies exist in the field of rehabilitation. Many studies have reported the positive impacts of VR on physical rehabilitation of different underlying diseases, including cognitive functions, balance and also the motor function of upper extremities. VR for motor rehabilitation has received a fair amount of attention, as the latest published review articles demonstrate [13,14,15]. VR has been proposed and utilized as an assistive rehabilitation technology for individuals suffering from cerebral palsy [16], cancer rehabilitation [17], spinal cord injuries [18], Parkinson’s disease [19], Guillain–Barré syndrome [20] and multiple sclerosis [21], among others. Furthermore, promising results have already been reported by studies regarding the benefits of VR-based rehabilitation for motor learning or the relearning of upper extremities after a stroke [22,23]. Three systematic reviews show that VR-based activities for upper extremity rehabilitation post-stroke were more effective when compared with traditional care [24,25,26].

Despite the proven potential of VR in neuromotor rehabilitation and a growing body of evidence on its effectiveness, the results initially showed a limitation in the evidence of improved upper extremity functionality beyond neurological conditions [27]. Nevertheless, the odds of a successful recovery are different for each disease, so the results obtained by VR interventions and their acceptance could be different as well. Lee et al. presented in their pilot study an innovative goal-directed shoulder rehabilitation system by combining VR techniques with wearable inertial measurement units to design rehabilitation exercises for patients with frozen shoulders and examine their effect on motor functions of the shoulder [28]. The results of the performance of each shoulder task showed significant improvements. The findings indicated that shoulder joint mobility improved significantly, which was also signified when performing shoulder strengthening exercises. The authors concluded that the patients´ shoulder muscle strength gradually increased with the progress of rehabilitation [28]. Another pilot study explored the effects of therapeutic immersive VR on pain in upper limb complex regional pain syndrome [29]. The study utilized the HTC Vive VR system, which consists of a wired headset, two handheld motion controllers and two base stations. VR therapy sessions consisted of guided visualization exercises and interactions with the virtual environment using their virtual hands. Eight participants were approved for up to ten sessions of VR therapy, with six fully completing the study. An immersive virtual three-dimensional interactive kitchen environment was designed that allowed visualization and manipulation of objects with virtual hands. Participants performed tasks representative of daily activities, as well as guided visualization exercises. Pre- and post-session pain scale measurements and subjective feedback were collected with each session. At the end of the study, participants reported a subjective improvement in their pain and daily functions. However, objective pain scales had limited correlation to the reported subjective relief. The authors concluded that immersive VR could potentially offer pain relief as well as functional improvement in selected patients with upper limb complex regional pain syndrome [29]. Moreover, varying results were obtained from several randomized controlled trials. The VR technologies in the included studies were rarely associated with measurable benefits related to different clinical outcomes. For example, the study of Parker et al. showed significant improvements in pain, favoring VR technology, while no statistically significant differences were detectable in the range of motion [30]. In contrast, the intervention group in the study by Joo et al. was associated with statistically significant improvements in pain perception [31]. Pekyavas et al. also obtained similar results, where, in terms of some parameters of functionality and pain perception, the group using VR showed superior results to the conventional rehabilitation group [32]. Further studies found no statistically significant differences at all with regard to upper extremity function or pain between participants using the VR technology and participants receiving traditional rehabilitation [33,34,35]. 

Interventions utilizing new technologies for rehabilitation should be analyzed for efficacy and the rehabilitants’, as well as therapists’, perspectives and experiences [36]. It is necessary to study both quantitative (e.g., the effectiveness of the intervention) and qualitative (e.g., rehabilitants’ acceptance of therapy) aspects regarding the application of new technology concurrently [37]. Before applying therapies based on novel technologies to larger sample sizes, these innovations must be progressively tested to describe their accuracy and acceptance in practice [36].

Therefore, the primary aim of this pilot study is to investigate the feasibility of a VR-based system for upper extremity injuries in outpatient rehabilitation, which is defined as the extent to which a new treatment or innovation can be successfully used or carried out within a given population or setting. Furthermore, little is known about the factors that might play a role for the sustainable implementation of VR as a complement therapy in the rehabilitation of upper extremity injuries as well as its use in the home environment from the perspectives of both rehabilitants and therapists. For this purpose, the study included quantitative and qualitative objectives. The objective of the quantitative design was to implement a novel VR technology and to analyze its effects on different clinical outcome measures in order to conduct further studies comparing different treatment alternatives. Additionally, by using a sociotechnical framework, namely the Unified Theory of Acceptance and Use of Technology (UTAUT I) [38], the acceptance and usability, referring to the participants´ perception and ability to use the system to achieve goals effectively and satisfactorily, were assessed as a qualitative objective in order to potentially prove the system and to provide a basis for iterative development and maintenance in the future. In this way, the efficiency and quality of the whole rehabilitation system can be improved and a reference can be provided for data-driven intelligent home rehabilitation.

## 2. Materials and Methods

This pilot study was conducted as a single-center trial over three weeks per patient. The inclusion criteria comprised patients over 18 years of age, a history of upper extremity injury and an indication for rehabilitation based on that history. Persons with degenerative or inflammatory diseases as well as neurological deficits and associated disturbances in normal movement, e.g., due to a stroke, were rejected. The eligibility of the participants was determined and documented with the help of a medical anamnesis at the beginning of the study by the study physician. In addition, therapists involved in the rehabilitation and supervision of the participants were included in the study. 

To ensure standardized instructions for the VR-based rehabilitation program, this was a mono-centric study and all participants were recruited from the outpatient rehabilitation center in Bielefeld. This study was approved by the Ethics Committee Westfalen-Lippe. This study was conducted in accordance with the Declaration of Helsinki, and all subjects gave written informed consent before participation.

### 2.1. The Intervention

The VR-based system in our study is intelligent software developed using the Unity game engine by Unity Technologies and the C# programming language. It integrates VR and motion-sensing technologies within a digital rehabilitation platform specifically designed for the treatment of rehabilitants with upper extremity injuries. The system consists of a head-mounted display connected to a portable computer and controllers equipped with motion detection sensors. In this study, we used Meta Quest 2 as the VR device to deliver the immersive experience. Users underwent three different exercises tailored for rehabilitants with upper extremity injuries, aiming to restore functional capacity. 

Our study utilized a virtual environment designed to immerse rehabilitants in a visually engaging room set in outer space. This environment features visually represented button objects that trigger user actions to initiate the games. Each exercise session is organized into five rounds per task, with each round lasting approximately 45 to 75 s, depending on the individual user’s performance. During the exercises, real-time feedback was provided to the rehabilitants through visual and auditory cues, informing them if the exercise was being executed correctly or if adjustments were needed. Furthermore, a summary of the user’s performance was displayed, providing feedback on their progress, and achievements were shown after each finished round. This structure encourages rehabilitants to strive for improvement and helps them track their rehabilitation progress throughout the session.

**Figure 1:**
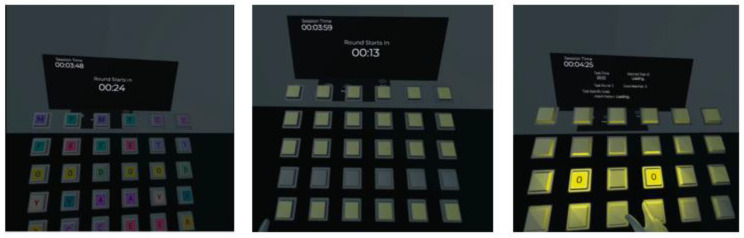
The memory buttons: the different stages of the first task in a three-panel layout. The left panel displays the uncovered alphabet letter pairs, providing an initial view of the task elements. The middle panel shows the covered letters just seconds before the exercise begins, setting the stage for the rehabilitant to engage in the memory task. The right panel captures the moment when a user successfully discovers and uncovers a pair of matching letters, highlighting the primary goal of the task.

The study encompassed three activities devised to promote rehabilitants‘ upper extremity movements. The first task focused on memory and involved matching button pairs. Rows of buttons containing alphabet pairs were generated before starting the task, and rehabilitants needed to remember the positions of each letter and its pair within the row. In this task, users extended their arms and reached for virtual buttons with their fingertips to reveal the alphabet letters. The exercise involves shoulder flexion and extension, elbow extension and flexion and wrist extension and flexion movements. These movements aim to enhance shoulder mobility, elbow joint functionality and wrist stability, contributing to overall upper extremity rehabilitation. Refer to Figure 1 for a detailed illustration of the gameplay in the first task.

**Figure 2:**
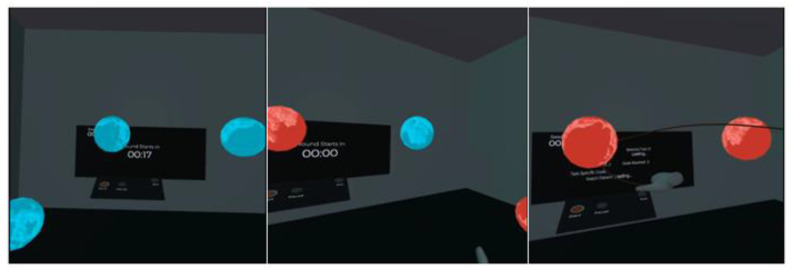
The star map: the different stages of the second task in a three-panel layout. The left panel displays the position of several available planets that can be connected during the exercise, providing an overview of the task elements. The middle panel shows two highlighted planets (in red) that are currently active and need to be connected, emphasizing the primary focus of the task. The right panel captures the user´s hand performing the action to connect the two highlighted planets, demonstrating the intended movement and interaction within the virtual environment.

In the second task, users were required to draw a three-dimensional trajectory between highlighted planets to connect them. The trajectories can take various forms, such as straight, diagonal or curved lines. Users must remember the positions of all the planets generated around them and draw the connecting lines accordingly. This exercise encourages users to engage in shoulder abduction and adduction and shoulder flexion and extension, as well as elbow flexion and extension. Additionally, this task stimulates wrist and forearm movements, such as pronation, supination and radial and ulnar deviation. The variety of possible trajectories enhances spatial awareness and motor control, making it an effective exercise for rehabilitants recovering from upper extremity injuries. Figure 2 provides a comprehensive visual overview of the second task environment and gameplay mechanics.

**Figure 3:**
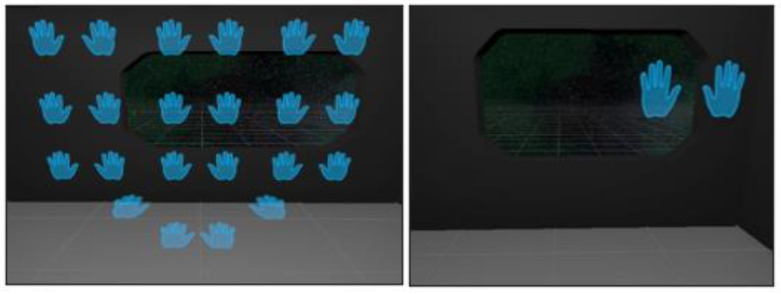
Forward-reaching movement pattern: The different stages of the third task in a two-panel layout. The left panel displays all the available positions of the pairs of hands, which are presented to the user seconds before the start of the exercise, providing an overview of the task elements. The right panel shows one pair of hands in the top-right position, ready to be matched by the user´s hand, demonstrating the intended movement of the task.

Inspired by the forward-reaching movement pattern applied by Thrasher et al. in their study [39], the third task challenges users to touch pairs of hands (left and right) from their respective sides. The left hand must reach the left-hand object, while the right-hand reaches the right-hand object. Both hands need to simultaneously reach the target hands within 0.5 s to proceed to the next pair of hands. In a single round, nine different target positions appear in random order, and the same pair does not reappear within the same round. This exercise promotes shoulder movement and elbow flexion for upper extremity rehabilitation while offering bimanual benefits. The simultaneous use of both hands enhances bilateral coordination and motor control, which are essential for performing daily activities. For a better understanding of the third task exercise layout and design, please see Figure 3.

A video demonstrating the virtual exercises and their implementation within the VR-based system can be found through the following link: https://drive.google.com/file/d/1nMFwr2W09qEcNx9cGL5MGJQI7UeS2WBD/view?usp=share_link (accessed on 10 May 2023).

Participants were enrolled in an alternate mode at the beginning of each program and were instructed in detail. At the end of the introduction phase, all participants received a manual, which was assembled in cooperation with physiotherapists and enabled participants to exercise with the VR system independently. According to the training schedule, every participant exercised daily in a total of 15 treatment sessions for approximately 30 min per session with the VR system. The training was integrated into their three-week outpatient rehabilitation program that was composed by the therapists according to participants conditions and needs. Besides the VR training, the training schedule of the participants consisted of gymnastics and ergotherapy, as well as educative elements for the management of upper extremity injuries. On principle, guidelines for outpatient rehabilitation of upper extremity injuries are already quite uniform in this setting, so that all participants had comparable elements of therapy irrespective of their indication for rehabilitation. An example of a daily training schedule is shown in Table 1.

### 2.2. Assessment and Statistical Analysis

A three-step assessment was performed before starting the VR-based rehabilitation (t0), at the end of the seventh treatment session (t1) and at the end of the 15th treatment session (t2). For each participant, the following outcome measures reflecting impairments were collected: the Disability of the Arm, Shoulder and Hand (QuickDASH), range of motion (ROM) of the upper extremity, health-related quality of life using the Short Form 36-Item (SF-36) and pain perception assessed on a visual analogue scale (VAS). In addition, user experience was assessed using the Suitability Evaluation Questionnaire (SEQ) for virtual rehabilitation systems and user acceptance was assessed through a self-developed questionnaire based on UTAUT I.

QuickDASH is a subset of 11 items from the 30-item DASH and is a self-reported questionnaire in which the response options are presented on a 5-point Likert scale. The questionnaire consists of eight function and three symptom items. At least 10 of the 11 items must be completed for a score to be calculated and the scores range from 0 (no disability) to 100 (most severe disability). This score was designed to be used for patients with any musculoskeletal disorder of the upper extremities [40]. QuickDASH was assessed at baseline (t0) and after the last training session (t2). 

ROM is characterized by the capability of a joint or body part to undergo its complete spectrum of movements. It is the measure of the distance and direction of movement around a specific joint or body part. A full range of motion is defined as the act of moving as far as anatomically possible during a given exercise [41]. In this study, goniometry measurements for joint ROM were taken at baseline (t0) and post-intervention (t2) by the involved therapists. Specifically, for each joint, the measured range was determined based on pre-study and post-study measurements [42]. All ROM measurements within the body segment (shoulder and elbow) were added to provide a composite ROM value. This value was then divided by the total number joint measurements taken, providing a mean ROM change per joint. The main joint movements were:

- Flexion: the bending of a joint. This decreases the angle between two body parts.
- Extension: the extending of a joint. This is a movement that increases the angle between two body parts.
- Abduction: movement away from the midline of the body.
- Adduction: the movement of a limb or other part towards the midline of the body or towards another part.

The SF-36 is a very popular instrument for evaluating the health-related quality of life. It comprises 36 questions that cover eight domains of health: physical functioning (PF), role physical (RP), bodily pain (BP), general health (GH), vitality (VT), social functioning (SF), role emotional (RE) and mental health (MH). Component analyses showed that there are two distinct concepts measured by the SF-36: physical dimension, represented by the Physical Component Summary (PCS) and a mental dimension, represented by the Mental Component Summary (MCS). A scoring algorithm is used to convert the raw scores into the eight dimensions listed above. The scores are transformed to range from zero, where the respondent has the worst possible health, to 100, where the respondent is in the best possible health [43]. The SF-36 was assessed at baseline (t0) and at t2. 

The VAS measures pain intensity. It consists of a 10 cm line, with two end points representing 0 (no pain) and 10 (pain as bad as it could possibly be) and was assessed at baseline (t0) and after the last training session (t2). 

The SEQ was used to assess the user experience with the VR technology. The SEQ was developed based on the short feedback questionnaire by Kizony et al. [44] and focuses on the suitability of the VR technology for the users. The questionnaire consists of 14 questions, of which 13 are rated on a 5-point Likert scale (question 1 to 11 ranging from 1 = not at all to 5 = very and question 12 and 13 ranging from 1 = very easy to 5 = very difficult). The 14th question is an open-ended question. The first seven questions relate to aspects such as usability, aesthetics and the sense of presence, as well as emotions related to the use, e.g., enjoyment and motivation of use. The following four questions relate to physiological reactions to the VR experience, such as discomfort or dizziness. Another two items deal with task-related aspects of the VR system and the general interaction with the system. The last question is open-ended and refers to possible reasons for perceived discomfort during the interaction. For the analysis of user experience, the mean values of the 13 questions were determined, which were then summed to a total score. The global score ranges from 13 points (poor suitability) to 65 points (excellent suitability). In its calculation, it is necessary to consider that items seven to ten as well as twelve and thirteen are negative items [45]. The SEQ was assessed at baseline after the first training session (t0), at t1 and at the last training session (t2).

**Figure 4:**
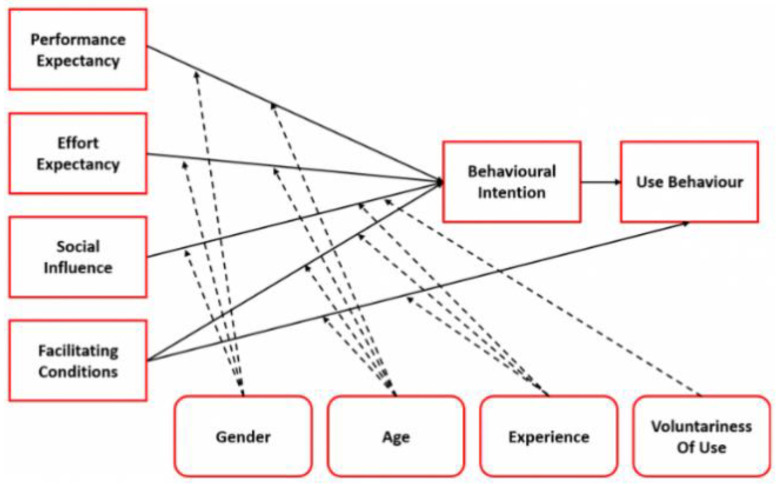
The Unified Theory of Technology Acceptance I (UTAUT I) [38].

In addition, a self-developed questionnaire based on UTAUT I was used to describe user experience [38] and was implemented at t1 and after the last training session (t2). UTAUT I is used to analyze user acceptance of information technologies and has already been used in studies with patients and health care professionals as well as validated in numerous studies in international social and technology science research [46,47,48]. As shown in Figure 4, actual behavior is dependent on behavioral intention, which is influenced by four determinants. Additionally, age, gender, experience and voluntariness of use moderate the relationship of the determinants and behavioral intention of use.

Except for the ROM, all questionnaires were filled out by the participants independently. The completion time was approximately 20 min per participant. Statistical analyses were performed using IBM SPSS v22.0 and Excel. Descriptive statistics were employed to describe the sample characteristics and outcome measures. 

### 2.3. Qualitative Data Collection

The qualitative research approach in its diversity is a currently accepted approach in order to understand the why and how of human actions. Both qualitative and quantitative methods complement each other. For this trail, the approach of a qualitative content analysis of interviews was chosen [49,50]. Semi-structured, face-to-face interviews were performed and recorded after the participants’ last training session. In addition, involved therapists were interviewed post-intervention. The decision to use a semi-structured interview technique was based on the opportunity for both the interviewer and the interviewee to discuss some topics in more detail. With open-ended questions, the rehabilitants and therapists were asked to explain their experience during the study period. Semi-structured interview guides were developed along the determinants of the UTAUT I differentiated for rehabilitants as well as therapists. The interview guides comprised three content-related complexes. The first complex consisted of questions about the experience with the VR-based system. The second complex was based on the determinants of UTAUT I, including questions regarding chances and challenges of VR-based systems for rehabilitation as well as barriers and facilitators for its implementation. The third complex dealt with expectations and suggestions for system improvement as well as immanent and exmanent follow-up questions. In order to check the comprehensibility of the interview guides and to identify problems in answering the questionnaire, three cognitive pre-tests were conducted with therapists and two with rehabilitants. 

All interviews were transcribed into written form. The analysis of interview material followed three steps: First, transcripts were organized and prioritized to identify issues and ideas that are relevant to the focus of the evaluation and in line with the categories of the interview guide in a deductive approach. Second, the words and phrases of each rehabilitant, which were used to describe the experience, were extracted and coded. In the last step, an inter-individual comparison was performed by structuring and grouping similar kinds of categories. In addition, categories were developed on the basis of the interview in an inductive approach. The codes and categories were discussed continuously by the study group in order to increase inter-individual consensus. Due to its nature, the qualitative data do not qualify for statistical analyses but are useful for the evaluation of feasibility and user acceptance.

## 3. Results

A total of six rehabilitants affected by upper extremity disease or injury were enrolled for study participation. One rehabilitant could not complete the rehabilitation program due to technical difficulties and was considered a dropout. Data analyses were performed on the five remaining subjects with complete data. Table 2 shows the demographic and baseline as well as post-intervention (t2) data of the rehabilitants enrolled in the study. Seeing as this paper is a pilot and has a small sample size (*n* = 5), the significance of results could not be calculated; therefore, only means and standard deviations were calculated and are presented. 

After 15 VR-based training sessions, the score in the two dimensions of the SF-36 increased. The parameter QuickDASH decreased over the study period. The pain perception measured on a VAS was on average slightly higher post-intervention compared to the baseline. In addition, Table 3 provides the average ROM from pre-study to post-study for each body area measured. Except for the extension of the shoulder, there was an increase in the ROM for all body areas measured from baseline to post-intervention. 

### 3.1. User Experience and Acceptance

Related to the user experience and the suitability of the VR-based system, SEQ shows moderate results (Table 4). The mean global score ranged from 44.4 after the first training session, to 50.4 after the seventh training session and finally to 45.2 after the last training session. Regarding particular items, rehabilitants did partly enjoy the experience with the system (Q1), were able to control the system (Q4) and did not feel confused or disoriented (Q10) in the course of the study. Three participants felt uncomfortable, since they could not exercise to the full extent due to technical difficulties. Altogether, the results indicate that after the seventh training session, the assessment was more positive compared to the assessment after the first training session. In the fifteenth training session, three participants had difficulties in performing the training to the full extent due to technical problems, where the therapists had to offer help. Consequently, it is clear that at the end of study period, the participants enjoyed using the VR system less than in the mid-term.

The assessment of user acceptance revealed important aspects of VR-based training for upper extremity rehabilitation during the study period. With regard to performance expectancy, the majority of rehabilitants disagreed that VR helped them to perform everyday tasks better during study period. In addition, they disagreed that their upper extremity functions improved over time. Regarding effort expectancy, most rehabilitants considered the VR-based system easy to handle and the training modalities understandable. In the domain of facilitating conditions, all respondents agreed to have the necessary abilities to operate the VR system and most of them agreed to have the knowledge required to use the system during study period. Furthermore, all respondents agreed with the statement that the integration of VR in conventional rehabilitation is possible and useful. In addition, all of them agreed with the aspect that professional support and guidance throughout training sessions with VR is highly important. With regard to social influence, the majority of rehabilitants confirmed the importance of their doctors’ or therapists’ attitude and recommendations towards VR-based training. In contrast, the opinion of relatives or friends is less important for rehabilitants’ own attitude formation and intention for use. Another major aspect for the use of VR is data protection regulation. For most of the rehabilitants, the confidentiality of their data was important. Lastly, almost all respondents agreed with the statement that they would consider VR for rehabilitation again and are openminded regarding use of VR systems in the future. The results of the acceptance analysis over the study period can be found in the Supplementary Materials (Figure S1).

### 3.2. Results of the Interview Analysis

In this pilot study, besides the five rehabilitants, four therapists (two males and two females) were interviewed. The results in the domain of performance expectancy provide insights into the perceived benefits of VR-based systems. According to the rehabilitants, the feedback from the interviews regarding the most frequent benefits of VR-based training were the following: to have more fun (*n* = 4) and to raise motivation due to animation (*n* = 4), as well as doing exercises that are in accordance with real life activities (*n* = 2). In agreement with the rehabilitants, all of the therapists indicated the playful design of therapeutical content is a significant advantage of VR-based systems, which could increase the motivation to carry out therapy and thus the rehabilitation success. Furthermore, they argued that the self-efficacy of the rehabilitants would be strengthened by the continuous and self-directed training with the VR system (*n* = 2). From the perspective of the rehabilitants, the fact of performing the exercises alone and not in interaction with a therapist was an important criticism of the VR-based training (*n* = 2). This aspect was also articulated by therapists (*n* = 2) and additionally the fact that movements might be performed incorrectly when not accompanied by therapists. Only two rehabilitants and one therapist emphasized that VR-based systems offer the advantage of exercising in their home environment and so they can avoid travelling long distances and conduct training sessions independent of the time and location. However, the majority of respondents could not imagine integrating VR-based systems for rehabilitation into their home environment at this point, as there are still uncertainties with regard to the system functions and effects. Motivational aspects and a lack of self-discipline to carry out the training sessions are also important aspects against the use of VR-based systems in the home environment, only from the perspective of the rehabilitants (*n* = 4). In particular, older respondents with a higher degree of physical impairments see themselves as less able to use VR systems on their own. 

Regarding effort expectancy, there were perceived difficulties regarding the usability of the system as well as the perceived potential negative effects of the training progress. Nearly all of the rehabilitants (*n* = 4) stated that when they were able to use the VR system, they could understand the contents and the exercises were easy to learn. However, it was essential that the system functioned appropriately and could be used regularly. It would be a considerable challenge if the screen and the system suddenly crashed or the game froze. In addition, all of the rehabilitants considered it as a barrier when the handling of the system was too complicated and incomprehensible. In this context, it is also problematic when the games are carried out outside the physical space due to the game design and thus creating a potential tripping hazard for the rehabilitants, which was articulated also by the therapists (*n* = 4). All of the therapists and the majority of the rehabilitants (*n* = 4) expressed that a VR-based system cannot be superior to conventional rehabilitation in terms of restoring upper extremity function. The respondents concordantly emphasized that a VR-based rehabilitation cannot replace conventional rehabilitation, but can only complement it. 

Information about the perceived structural and organizational conditions might have a positive effect on the intention to use the VR-based system. The rehabilitants reported that the instructions and support provided by the therapists were of high quality both in the beginning of the study and later in the subsequent VR training sessions (*n* = 3). Here, the rehabilitants again highlighted the importance of continuous support and guidance by therapists to correct movement execution as well as resolve unforeseen occurrences in handling the system (*n* = 4). In this context, the therapists agreed with rehabilitants and expressed the importance for rehabilitants to have a qualified contact person to solve therapeutical as well as technical problems, which is connected to the requirement of the therapists to receive sufficient instruction and training to deal with the VR-based system and acquire new competencies (*n* = 3). Moreover, when VR-based systems are going to be used in the long term in the context of rehabilitation, it must be ensured that the technical equipment can be adequately stored in the rehabilitation facilities. Ideally, it should be possible to have a lockable room or space to set up the equipment (*n* = 4). In addition to personnel and organizational requirements, however, monetary and legal conditions also play a role in the implementation of VR-based systems for rehabilitation. The therapists stated that the implementation of new technologies in organizations is generally associated with high costs and ambiguous legal requirements, so it is necessary to clarify questions with regard to liability or responsibility before the new system is actually used (*n* = 2).

The social influence domain comprises the relevance of subjective perceptions about explicit attitudes and views of third parties for intention to use. With regard to this, all of the therapists articulated that the attitudes of colleagues as well as the private environment would not shape their own opinion. Four rehabilitants expressed also that the attitude of important third parties, e.g., family members and friends, would not have an influence on their own attitude or their intention to use the system. Two respondents stated that positive attitudes or the recommendations of professional authorities would, however, influence their intention to use and their attitude towards VR-based systems. 

When asked what design suggestions the participants would have for future VR systems, one suggestion of the rehabilitants (*n* = 3) as well as therapists (*n* = 2) was that the continuous therapeutical support of the rehabilitants by a virtual therapist could help to establish therapeutic alliance. Furthermore, it was stated by rehabilitants (*n* = 4) and therapists (*n* = 2) that the possibility to choose from a variety of exercises according to need and preferences was important. A greater variation in exercises that can be adapted to specific circumstances, e.g., age, previous experience or physical ability, could increase the motivation to train. In addition, it was important for therapists (*n* = 2) to document the therapy progress and achieved therapy goals, as well as the rehabilitants (*n* = 2), in order to be able to align further therapy and maintenance motivation. With regard to technical equipment and hardware, two rehabilitants and three therapists stated that wireless head-mounted-displays would be easier to handle and that the computer performance and graphic cards would have to be adapted to enable smooth functioning. In addition, it should be ensured that the users have easy access to the system and the training sessions. 

Despite all the criticism and disadvantages, the majority of rehabilitants (*n* = 4) and all therapists confirmed the general feasibility and easy handling of the VR-based system and were willing to use VR-based technologies for rehabilitation purposes, provided that VR-based training is integrated as a complementary component in conventional rehabilitation training.

## 4. Discussion

The aim of this pilot study was to analyze the feasibility as well as the acceptability and implementation of a VR-based system in the context of upper extremity rehabilitation beyond neuromotor rehabilitation with a multi-perspective approach. For this reason, experiences and expectations as well as conditions related to the implementation of a VR-based system from the perspective of rehabilitants and therapists were assessed using a sociotechnical framework. 

In line with previous studies [51,52], the results of functional outcomes show that such an approach might be beneficial to rehabilitants, since improvements to measured parameters were observed, even if no significant changes could be shown. With regard to user experience and suitability of the VR-based system, a variety of aspects could be identified that provide information about the considerable potential for optimization.

In particular, following the participants’ reported advantages of the VR-based system in the interviews, it could be a valuable opportunity for additional physiotherapy-like exercises for interested rehabilitants. The rehabilitants as well as the therapists suggested that VR offered an enjoyable way to exercise and that this could motivate people to engage in rehabilitation programs. These experiences appear to reflect those described by Brady et al. [53], who pointed out, as a result of focus groups with physiotherapists, how most participants smiled when they described their experience of using VR, because it is fun. For neurological rehabilitation that often involves extensive repetition of basic tasks, VR has been shown to provide an engaging platform for rehabilitation by making therapy more stimulating [54,55]. Warland et al. explored the feasibility and acceptability of using VR for upper extremity rehabilitation following a stroke from the perspective of the rehabilitants and found that VR was a source of motivation for participants [55]. They described how the concept of “time flying” was positively correlated with enjoyment of VR and occurs when a participant is immersed in a goal-directed task in VR. Due to the nature of VR, we in addition believe that the positive results of this study obtained in a short period of training were due to a high level of active participation of rehabilitants during training, and also due to the possible comfort of specific training [56]. In further studies, it would be interesting to assess the engagement of the rehabilitants during training [57]. 

The therapists in this study did not report worrying about being replaced in their professional role by VR-based systems. Indeed, the implementation of VR in the context of rehabilitation, as well as the daily work routine, can have added value if there is a therapeutic benefit facilitated by the use of VR. The identified role of therapists in VR-based training is consistent with new upcoming self-management concepts that emphasize the empowerment and self-efficacy of rehabilitants [58]. Additionally, the aspect of interpersonal interaction will still play a major role in rehabilitation success, so that the use of VR might be considered as a complementary training option [59,60,61].

Therapists required improvements in VR characteristics to align better with the activities of relevant daily life tasks and therapeutical goals. Rehabilitants expressed their expectations of VR characteristics, which include hardware that functions properly and that adapts to human movements. Improved interprofessional cooperation and knowledge exchange between engineers of VR-based devices and therapists as experts on human movements and patient-centered needs in daily living could reduce the current gap, which is also supported by the findings of Tatla et al. [62]. The therapists also acknowledged that it took time to help and instruct rehabilitants to navigate the VR system. They also felt that VR may suit rehabilitants who were already tech-savvy and considered that younger rehabilitants may have greater success with using VR. However, they felt that with further training and instruction, they would acquire the competencies for delivering VR-based exercises to their rehabilitants. 

In addition, the rehabilitants expressed the desire to adapt training sessions offered by VR to their individual needs and requirements. The VR system used in this study offered three different movement exercises. This was sufficient for the use in this pilot study, but feedback from the participants indicated that more variation in training modalities would be beneficial for long-term use. Another way to design training sessions with VR is to add a recording component. This might be a way to involve therapists in the exercises in such a way that they are able to specify certain movements for individual rehabilitation goals. For example, therapists can use the VR system in a demonstration at the beginning of rehabilitation and record the movements as a sort of choreography. These recorded sequences can then be played back and visualized in the form of a photofit picture by the rehabilitants during their subsequent exercises. In this way, rehabilitants could orient their movements as closely as possible to this photofit picture, which on the hand, enables an individually focused and challenging training and, on the other hand, ensures the correct execution of certain movements. The concern that VR could lead to incorrect movement execution as well as symptom aggravation due to the uncontrolled nature of exercise has also been expressed in the study of Brady et al. [53]. In addition, Kelly et al. [63] reported that participants with chronic low back pain who used a VR intervention experienced muscle soreness and back pain that they attributed to an increase in movement and muscle use while exercising with VR. Currently, there is little evidence that using VR causes injury or pain, but this must be explored further in people with musculoskeletal diseases and respective upper extremity injuries. 

Of course, the analysis of the interview material revealed also points of criticism as well as disadvantages of the VR-based training. 

In this study, therapists as well as rehabilitants expressed significant concern about the safety of using immersive VR, especially in a non-supervised manner. Therapists were worried that rehabilitants might fall and injure themselves because of the immersive nature of VR. They felt that rehabilitants may not pay attention to safety features such as virtual boundaries and bump or trip over objects in the real world. Much of the literature on VR interventions for musculoskeletal rehabilitation uses non-immersive technology, which allows the user to see the real-world environment, therefore reducing the risk of accident or injury. A recent review that explored the effectiveness of immersive VR interventions for managing musculoskeletal pain reported the frequency and nature of adverse events [64]. In this review, no adverse events that involved accident or injury were reported; however, many of the interventions were carried out in a supervised manner or in a seated position. None of the included studies involved whole body standing exercises in an unsupervised setting.

The participants reported several times that they currently cannot imagine training with VR in their home environment, since there are still uncertainties regarding function as well as ambiguous legal circumstances. Nevertheless, research indicates that adults can use VR technology successfully at home. A systematic review by Miller et al. [65] investigated the effectiveness of VR interventions for enabling physical activity in older adults and people with neurological conditions. As a secondary aim, they explored the feasibility of using VR in this cohort and found high levels of retention and adherence across studies [65]. Impressions of the easiness of the VR training are in contrast to their apprehension that human movement is more complex and consequently difficult to illustrate in VR. Compensating for the reported lack of sensory input in VR training sessions will be challenging in future VR development. Further questions regarding VR training benefits and its meaningfulness have emerged. This underlines the need for further research on how to best develop and implement this approach in rehabilitation.

The findings need to be assessed taking into account some limitations. Being a pilot study, the results should be interpreted with caution and regarded as a preliminary work, as more data are necessary to confirm the results. The main limitation of this study is related to the small sample size. Despite extensive recruitment efforts, only five rehabilitants—consisting mainly of males who were on average older than 40 years—could be included in the study who met the inclusion criteria. Additionally, it is important to consider that a study period of 15 training sessions (15 days) per rehabilitant is rather short to measure potential improvements in functional capacity. Moreover, study participants did not only exercise with the VR system but underwent further therapy modalities, so that positive results cannot be solely attributed to the VR system. Furthermore, there was no control group and no rehabilitants with different underlying upper extremity injuries were included, so the comparability of the results is limited. Another limitation of the current study is that all of the participants were based in one rehabilitation center located in one specific region. Their experiences and perspectives on the potential role of VR in rehabilitation may be influenced by both their clinical and cultural backgrounds. It is possible that participants from different establishments or regions could have different views; therefore, results from this study cannot be generalized. Rather less relevant, but not to be excluded, are biases caused by the phenomenon of social desirability [66]. Although the participants were assured of anonymity, it might be possible that participants responded in a socially accepted manner. Overall, taking into account the sample composition, the results should not be considered as representative, but rather a reflection of trends and patterns of attitudes from different perspectives. 

Taking all this together, this study sought to explore on the one hand the feasibility of a VR intervention in a specific clinical setting for the rehabilitation of upper extremity injuries, as well as to explore attitudes towards the role of VR in rehabilitation from a multi-perspective approach. We included both rehabilitants and therapists, as postulated in existing research [53], to get a full picture of the potential of and barriers to using VR in clinical practice for this population and to inform the future development of VR applications [67]. Future development of VR systems will benefit from an interprofessional collaboration between rehabilitants, therapists and engineers. Concurrently, training concepts should be developed with the aim of addressing rehabilitants’ needs in daily living. Additionally, it should be investigated, e.g., in small clinical trials, to what extent VR-based rehabilitation differs from conventional rehabilitation in terms of clinical efficacy and acceptability as well as implementation in a home environment before investing in large-scale randomized controlled trials. 

## 5. Conclusions

Altogether, the results of this study suggest that while VR was seen as an enjoyable and exciting complement to rehabilitation and potentially opened up new ways for upper extremity rehabilitation, its novelty raised some concerns and apprehension. The findings provide valuable insights relating to the feasibility and usability of a VR system as well as into rehabilitants’ and therapists’ acceptance of it as a complement to rehabilitation. However, further research is needed to figure out how a human-centered design of VR can contribute to upper extremity rehabilitation. 

## Figures and Tables

**Table 1:** Exemplary daily training schedule.

Time Frame	Training Modality
At 9:00 a.m.	Heat therapy
At 9:30 a.m.	Medical training therapy
At 10:30 a.m.	VR training
At 11:00 a.m.	Lunch break
At 11:30 a.m.	Ergotherapy
At 12:30 p.m.	Progressive muscle relaxation
At 13:00 p.m.	Rest time
At 14:00 p.m.	Motion bath

**Table 2:** Demographic and clinical assessment of sample at baseline and post-intervention (*n* = 5).

Variable		N
Gender	F:M	2:3
Age	Category	31 to 40 years: 1
		41 to 50 years: 2
		51 to 60 years: 1
		Over 60 years: 1
Level of education	Category	Lower-level employees: 1
		Upper-level employees: 4
Indication for rehabilitation	Category	Following humeral fracture: 3
		Following injury of the shoulder: 2
Site of lesion	Category	Left: 0
		Right: 5
Previous VR use	Category	Yes: 2
		No: 3
QuickDASH	Mean ± SD	
Baseline		49.55 ± 7.08
Post-intervention (t2)		44.55 ± 8.29
SF-36_MCS	Mean ± SD	
Baseline		41.10 ± 17.07
Post-intervention (t2)		43.36 ± 14.49
SF-36_PCS	Mean ± SD	
Baseline		39.52 ± 4.54
Post-intervention (t2)		42.04 ± 7.09
VAS	Mean ± SD	
Baseline		2.60 ± 2.51
Post-intervention (t2)		3.00 ± 2.92

**Table 3:** Mean ROM (degree) at baseline and post-intervention (*n* = 5).

Body Area	Movement Measured	Mean ± SD
Shoulder	Abduction baseline	89.00 ± 24.60
	Abduction post-intervention (t2)	115.00 ± 32.79
Shoulder	Extension baseline	42.00 ± 28.85
	Extension post-intervention (t2)	34.00 ± 11.40
Shoulder	Flexion baseline	98.00 ± 56.30
	Flexion post-intervention (t2)	133.00 ± 27.30
Elbow	Extension baseline	6.00 ± 8.22
	Extension post-intervention (t2)	5.00 ± 4.47
Elbow	Flexion baseline	97.00 ± 68.34
	Flexion post-intervention (t2)	146.00 ± 8.94

**Table 4:** Results of the SEQ in study period (*n* = 5).

Question	Mean ± SD after the First Session	Mean ± SD after the Fifth Session	Mean ± SD after the Fifteenth Session
Q1: How much did you enjoy your experience with the system?	2.8 ± 1.79	3.4 ± 0.55	2.2 ± 1.30
Q2: How much did you sense to be in the environment of the system?	3.2 ± 1.30	3.6 ± 0.89	2.6 ± 1.52
Q3: How successful were you in the system?	2.8 ± 1.64	3.6 ± 1.14	2.8 ± 1.79
Q4: To what extent were you able to control the system?	3.0 ± 1.87	4.0 ± 1.23	2.6 ± 1.52
Q5: How real is the virtual environment of the system?	3.2 ± 1.09	3.8 ± 0.84	2.6 ± 1.52
Q6: Is the information provided by the system clear?	2.8 ± 1.64	4.00 ± 1.00	2.8 ± 0.84
Q7: Did you feel discomfort during your experience with the system?	2.4 ± 1.14	1.8 ± 1.82	1.0 ± 0.06
Q8: Did you experience dizziness or nausea during your use of the system?	1.0 ± 0.06	1.0 ± 0.06	1.0 ± 0.06
Q9: Did you experience eye discomfort during your use of the system?	1.2 ± 0.45	1.4 ± 0.89	1.0 ± 0.06
Q10: Did you feel confused or disoriented during your experience with the system?	1.8 ± 1.30	1.0 ± 0.06	1.0 ± 0.06
Q11: Do you think that this system will be helpful for your rehabilitation?	1.8 ± 1.09	3.0 ± 1.03	2.4 ± 1.34
Q12: Did you find the task difficult?	2.4 ± 1.01	2.6 ± 0.55	2.4 ± 1.67
Q13: Did you find the devices of the system difficult to use?	2.4± 1.01	2.4 ± 1.04	2.4 ± 1.67
Q14: If you felt uncomfortable during the task, please indicate the reasons.	Open response		
T: Global Score	44.4 ± 2.61	50.4 ± 1.27	45.2 ± 2.19

## Data Availability

Not applicable.

## Supplementary Materials

The following supporting information can be downloaded at: https://www.mdpi.com/article/10.3390/healthcare11101498/s1, Figure S1: Acceptance analysis based on the Unified Theory of Technology Acceptance I (UTAUT I).
