# Supplementary material for: Virtual Reality for Upper Extremity Rehabilitation—A Prospective Pilot Study
Source: Healthcare (Basel). 2023 May 21;11(10):1498. doi: 10.3390/healthcare11101498 (PMC10218592; doi:10.3390/healthcare11101498)
Supplement: Supplementary file 1 [file healthcare-11-01498-s001.zip › healthcare-2341125-supplementary.pdf]

Figure S1: Acceptance analysis based on the Unified Theory of Technology Acceptance I (UTAUT I)

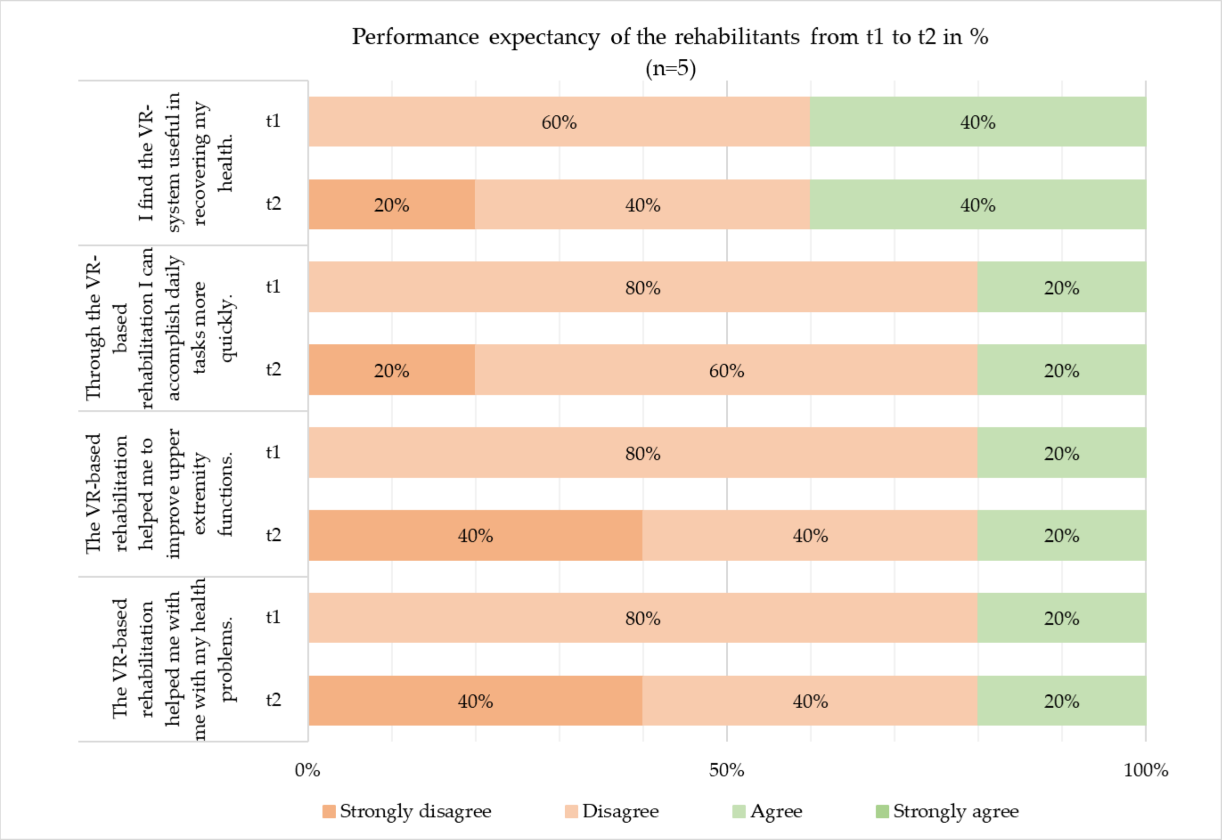

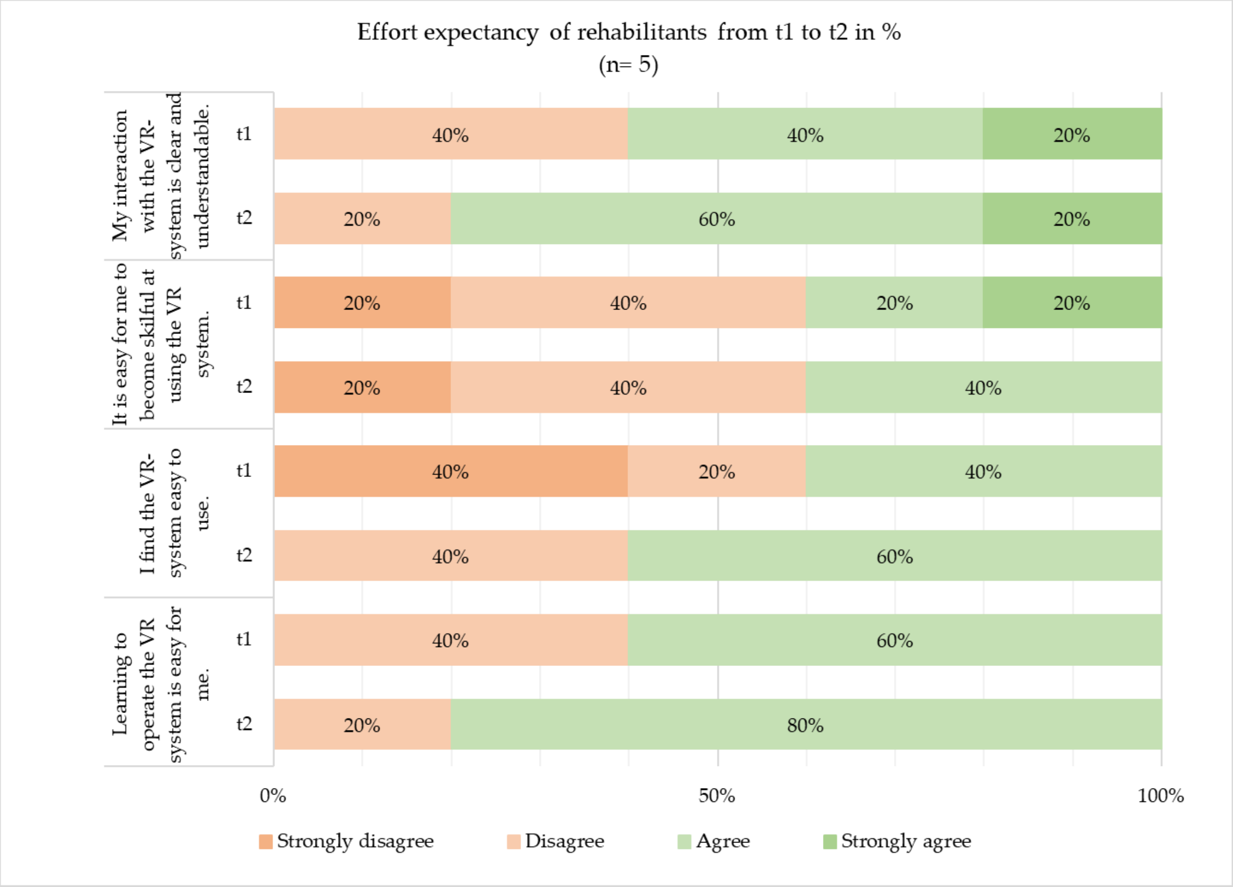

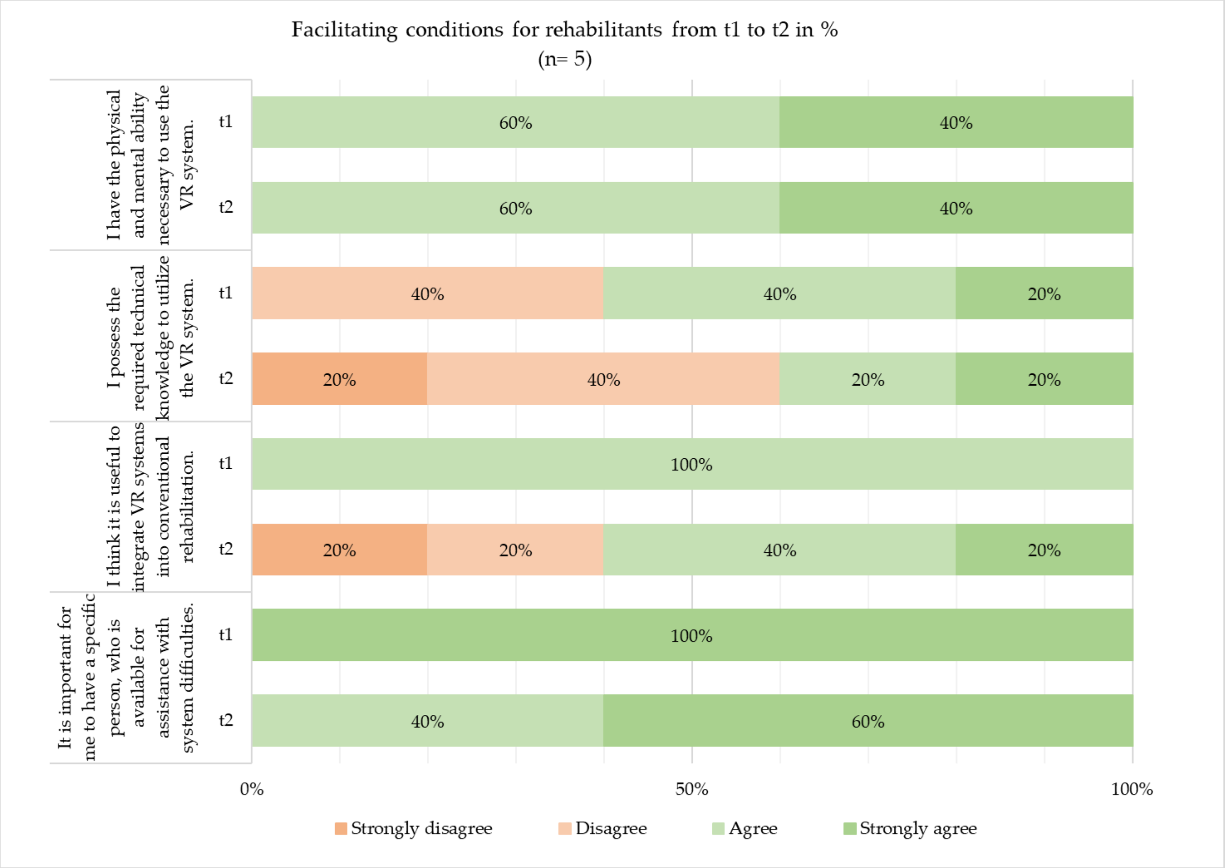
Facilitating conditions for rehabilitants from t1 to t2 in %
(n= 5)
I have the physical and mental ability necessary to use the VR system.
t1
60%
40%
t2
60%
40%
I possess the required technical knowledge to utilize the VR system.
t1
40%
40%
20%
t2
20%
40%
20%
20%
I think it is useful to integrate VR systems into conventional rehabilitation.
t1
100%
t2
20%
20%
40%
20%
It is important for me to have a specific person, who is available for assistance with system difficulties.
t1
100%
t2
40%
60%
0%
50%
100%
Strongly disagree
Disagree
Agree
Strongly agree

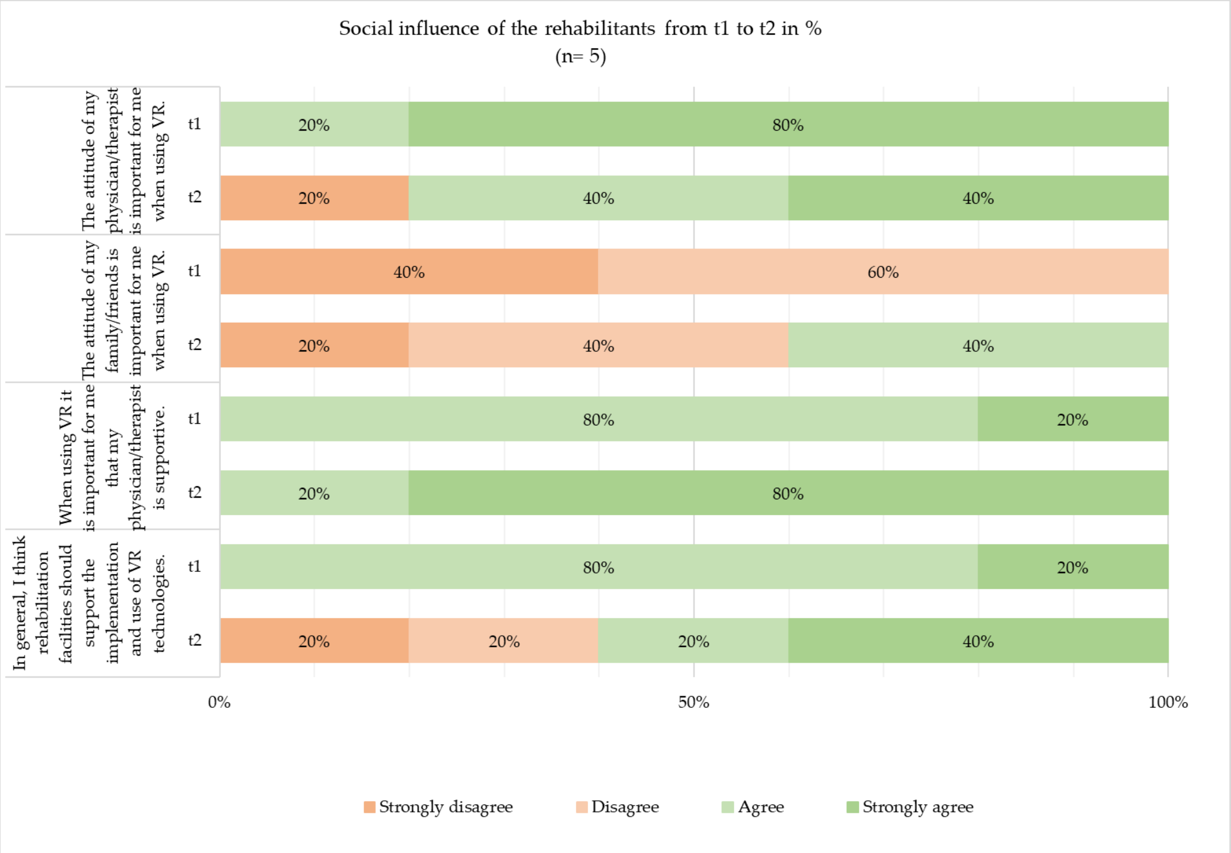
Social influence of the rehabilitants from t1 to t2 in %
(n= 5)
The attitude of my physician/therapist is important for me when using VR.
t1
20%
80%
t2
20%
40%
40%
The attitude of my family/friends is important for me when using VR.
t1
40%
60%
t2
20%
40%
40%
When using VR it is important for me that my physician/therapist is supportive.
t1
80%
20%
t2
20%
80%
In general, I think rehabilitation facilities should support the implementation and use of VR technologies.
t1
80%
20%
t2
20%
20%
20%
40%
0%
50%
100%
Strongly disagree
Disagree
Agree
Strongly agree

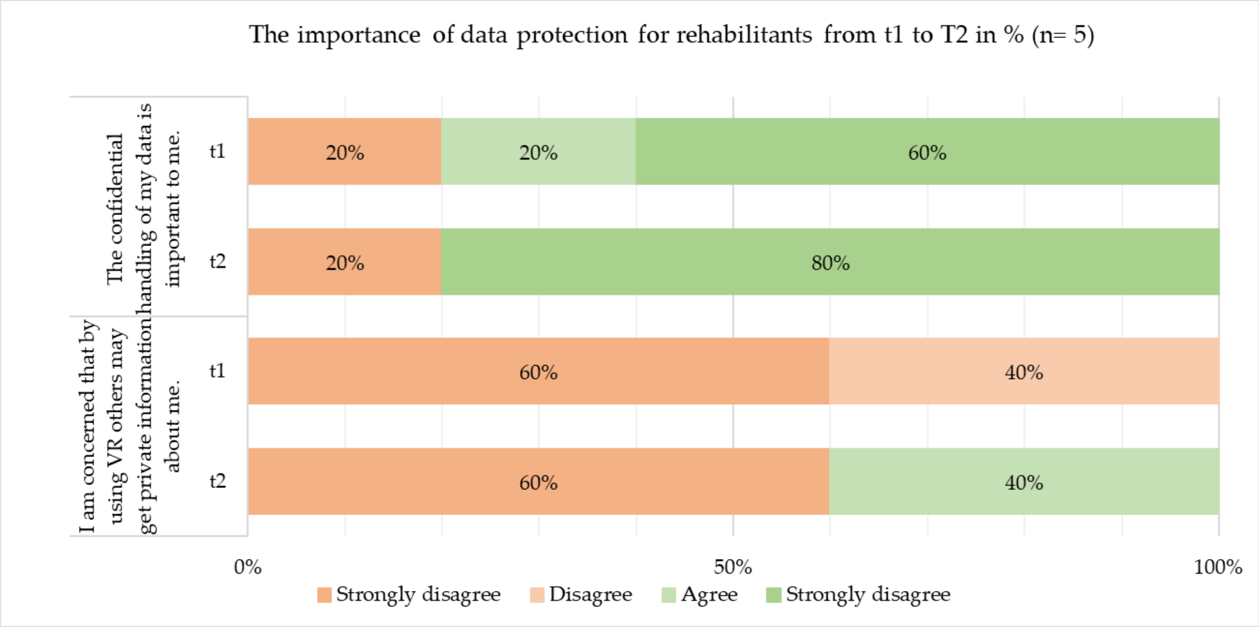

The importance of data protection for rehabilitants from t1 to T2 in % (n= 5)
The confidential handling of my data is important to me.
I am concerned that by using VR others may get private information about me.
t1
t2
20%
60%
80%
40%
0%
50%
100%
Strongly disagree
Disagree
Agree
Strongly disagree

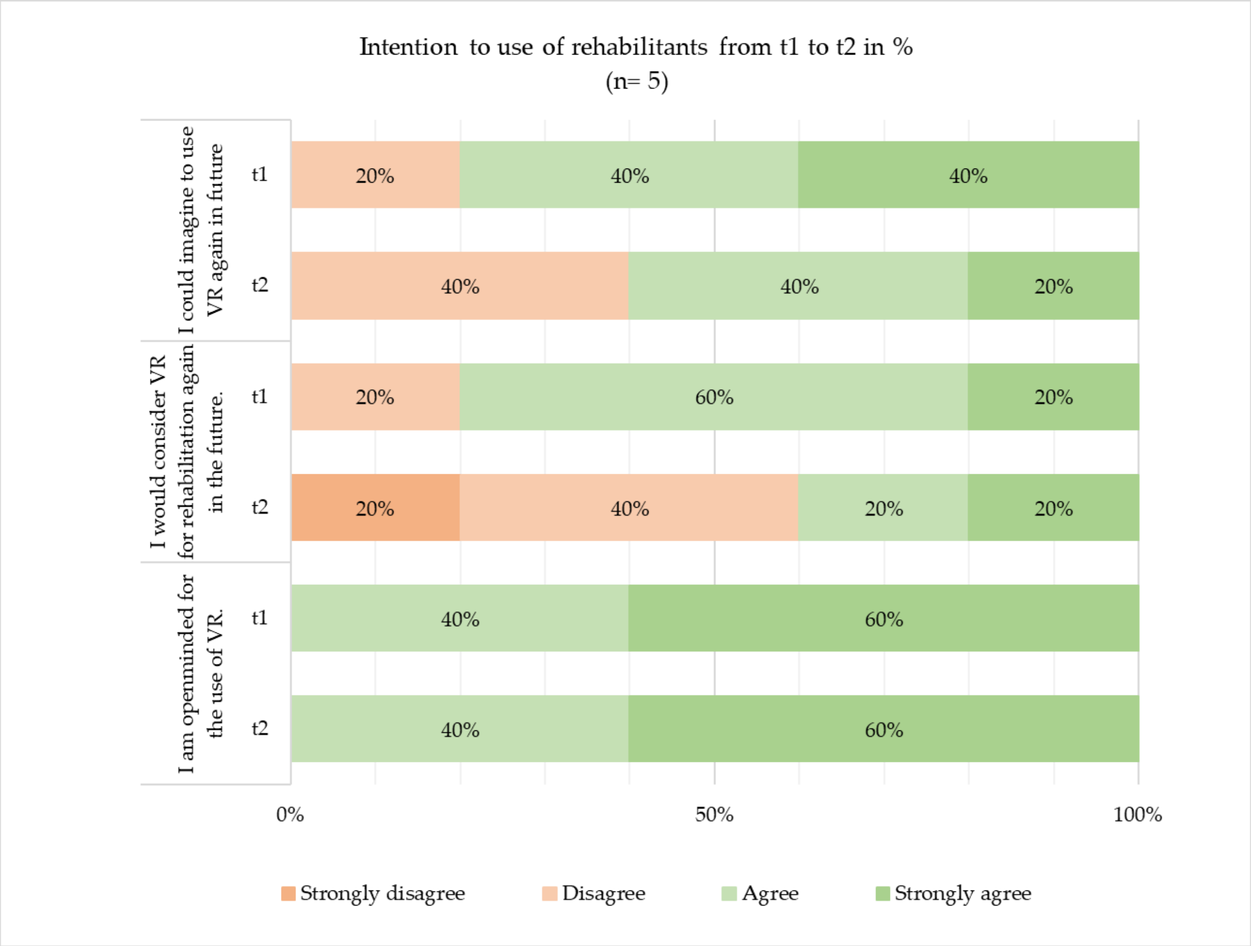

Intention to use of rehabilitants from t1 to t2 in %
(n= 5)
I could imagine to use VR again in future
I would consider VR for rehabilitation again in the future.
I am openminded for the use of VR.
t1
t2
20%
40%
60%
0%
50%
100%
Strongly disagree
Disagree
Agree
Strongly agree
